# New advancements in the management of Neuromyelitis Optica spectrum disease: literature review

**DOI:** 10.3389/fopht.2023.1130971

**Published:** 2023-05-16

**Authors:** Padmaja Sudhakar, Khawla Abusamra, Mangayarkarasi Thandampallayam, Ashwini Kini

**Affiliations:** Department of Neurology, University of Kentucky, Lexington, KY, United States

**Keywords:** Neuromyelitis Optica spectrum disorder, Eculizumab, Satralizumab, Inebilizumab, therapy

## Abstract

Neuromyelitis Optica spectrum disorder (NMOSD) is a relapsing autoimmune disease of the central nervous system (CNS) where aquaporin-4 water channels are the antigenic target of the disease. The spectrum of the disease involves regions of the CNS where the water channel is widely expressed including the spinal cord, the optic nerve, dorsal medulla, brainstem, and thalamus/hypothalamus. Management of NMOSD includes acute as well as long term treatment. Acute symptoms are typically treated with intravenous corticosteroids and/or plasma exchange while long-term treatment involves the use of immunosuppression/immune modulation. The year 2019 is thought to be the “year of the NMOSD” as three new medications became available for this devastating disease. In this review, FDA approved NMOSD medications are discussed.

## Introduction

Neuromyelitis Optica spectrum disorder (NMOSD) is an autoimmune relapsing disease of the brain and spinal cord where aquaporin-4 (AQP4) water channels are the main antigenic target (1). The disease involves regions of the central nervous system (CNS) where water channels are widely expressed. The diagnostic criteria for NMOSD incorporate six clinical presentations involving different areas of the CNS including the spinal cord causing longitudinally extensive transverse myelitis (LETM), the optic nerve causing optic neuritis (ON), the medulla causing area postrema syndrome, the brainstem causing acute brainstem syndromes, and the thalamus/hypothalamus causing acute diencephalic syndromes (1, 2). The occurrence of one of the above-mentioned syndromes in the presence of serum AQP4-IgG + autoantibodies makes the diagnosis of AQP4-IgG+ NMOSD (2).

As NMOSD attacks can lead to significant long-term disability, pharmacotherapy aims at aggressively treating acute inflammatory attacks and prevent future attacks to reduce CNS damage and preserve neurologic function (3, 4).

The management of NMOSD includes acute as well as long term treatment (3). Acute attack is typically treated with high dose intravenous corticosteroids in the form of 1 gram of methylprednisolone daily for 5 days followed by gradually tapered doses of oral prednisone, typically over months in selected cases. If improvement is not seen within days of corticosteroids, plasma exchange (PLEX) or immunoadsorption are possible options (3–5) Some experts in the field suggest the use of PLEX or immunoadsorption as a first line treatment option in the treatment for subsequent attacks, especially for LETM (4, 5).

NMOSD is a devastating disease where if patients are left untreated will suffer Long term complications (1, 2) Many medications have been used for long term immunosuppression such as Rituximab, Mycophenolate, and Azathioprine with variable success rates (3). Most recently, three monoclonal antibodies (**Table 1**) have been approved by the United States Food and Drug Administration (FDA) for the treatment of NMOSD. The year 2019 is thought to be the “year of the NMO” when 3 new drugs became available as treatment options for this otherwise devastating disease (3). In this paper, FDA approved medications for adult onset NMOSD will be reviewed in detail.

**Table 1:** FDA approved medications for NMOSD.

Medication	Mechanism of Action	Clinical Trial/s	Recommended dosage	Reported side effects
Eculizumab	C5 monoclonal antibody	PREVENT trial	900 mg every week for initial four doses, followed by 1200 mg every 2 weeks	Meningococcal infection, upper respiratory tract infection, headache, nausea, nasopharyngitis.
Satralizumab	Blocks IL-6 signaling pathway	Sakurastar and Sakurasky trial	Initial injection of 120 mg, followed by another injection at week 2 and week 4 as loading doses, followed by a maintenance dose of 120 mg every 4 weeks	headache, arthralgia and injection site reactions.
Inebilizumab	Anti-CD19 monoclonal antibody	Phase II-III N-Momentum trial	Intravenous infusion with 2 initial doses of 300mg separated by 2 weeks and followed by 300mg every 6 months	infection and infusion related reactions

### Eculizumab

Eculizumab is a humanized monoclonal antibody that has been tried in NMOSD with favorable results. Eculizumab is a terminal complement inhibitor that prevents the C5 convertases from associating with C5, which in turn prevents C5 cleavage to the proinflammatory complement component C5a and C5b (6). It has been used in paroxysmal nocturnal hemoglobinuria, atypical Hemolytic uremic syndrome as well in refractory seropositive generalized Myasthenia Gravis. AQP4 IgG has shown in cell-based assays to induce a potent complement mediated toxicity, which could likely explain the utility of a complement inhibitor in treatment of NMOSD. The recommended dose of Eculizumab is 900 mg every week for initial four doses, followed by 1200 mg every 2 weeks (6). Early studies of this drug by Pittock et al. on 14 patients showed reduced relapse in AQP4-IgG+ NMOSD (6). Pharmacokinetics data have shown that Eculizumab achieves steady state concentration by about week 4 and thereon, complement inhibition is noted by Day 1 of infusion through the pre-dosing trough levels, thus suggesting early and sustained action of the drug (7). The PREVENT trial was a multicenter, randomized, double-blind, time-to-event trial from 18 countries where 143 adults were randomized to receive either intravenous eculizumab or placebo. The study included AQP4 IgG positive patients who had 2 attacks in previous 6 months (8) The annualized relapse rate in the past 24 months prior to initiation of Eculizumab was comparable between both groups (1.94 vs 2.07) (8). Transverse myelitis was the most common followed by optic neuritis then brainstem and cerebral symptoms (6). On comparison of the annualized adjudicated attack rate between the 2 groups at the end of the study the Eculizumab group was 0.02 in contrast to the placebo group of 0.35. The first adjudicated attack was seen in 3% of the Eculizumab group and 20% of the placebo group. Per study results “The proportions of eculizumab- and placebo-treated patients who remained attack-free were 97.9% and 63.2% respectively at week 48, 96.4% and 51.9% respectively at week 96, and 96.4% and 45.4% respectively at week 144” (7, 8). The attack free rate was lower (more attacks noted) in the subgroup of the placebo cohort that was not on any other immunosuppressive agent (6). A subgroup analysis on the Asian population included in the study suggested Eculizumab was equally effective with a similar side effect profile in this subgroup and this similar efficacy was noted in the *post hoc* subgroup analysis across different age group, races, sex as well as geographic location (9).

Upper respiratory tract infection, headache, nausea, nasopharyngitis were some of the reported side effects from the study. The most important precaution is initiating meningococcal vaccination (against serogroups ACWY and B) prior to starting on Eculizumab. It is recommended that patients receive the required booster vaccination for meningococcal meningitis for the duration that they are on Eculizumab as there is significant 1000-fold increase in risk of meningococcal disease in comparison to the general U.S. population due to complement factor inhibition which are essential components against fighting the meningococcal bacteria (6). Though the advisory committee on Immunization practice recommendation in Eculizumab is for meningococcal vaccination only, there is theoretical risk for increased infection from encapsulated organisms, hence clinicians need to consider vaccination for H. Influenza and Pneumococcal infections. It is recommended to receive antibiotic prophylactic coverage if the infusion was initiated in less than 14 days of having received the vaccination (6, 8). Between 2006-2018, there were a total of 16 reported cases of meningococcal disease in patients receiving Eculizumab, 14/16 who had received the vaccination. It was however noted that 11/16 were non-groupable (10).

However, the study involved only AQP4-IgG seropositive patients and we are unable to extrapolate the same results for seronegative patients. The study extended into an open label study after completion of the short 6 week follow up when the trial was terminated early. On the open label extension, per the paper “At 192 weeks (4 years), 96% patients were adjudicated attack free. 95% (20/21) of patients on Eculizumab were stable and had no disability worsening thus providing effective long-term relapse prevention, making it a desirable drug in patients requiring long term therapy for this disabling disease” (6, 11). Relative to placebo, per the paper “Eculizumab showed significant reduction in the annualized rate of adjudicated attack-related hospitalization (0.012 vs. 0.267; *p* < 0.0001), use of IV methylprednisolone (0.012 vs. 0.286; *p* < 0.0001), high-dose oral corticosteroids (0.012 vs. 0.114; *p* = 0.0021) and PLEX (0.012 vs. 0.134; *p* = 0.0006)” (6, 11).

Some questions that remain are the effect from concomitant use of immunosuppressive agents in almost 70% of patients, efficacy of the agent in seronegative patients, and safety profile in pregnant women as currently there is no available data on the same.

Ravalizumab is a C5 complement inhibitor with a similar mechanism as Eculizumab, the efficacy and safety of which was reported in a phase III clinical trial, the CHAMPION-NMOSD. The study revealed that Ravalizumab significantly reduced the relapse rate while maintaining a good safety profile. The main advantage over Eculizumab was longer dosing interval as compared to Eculizumab due to longer half-life (12).

### Satralizumab

Satralizumab is a humanized immunoglobulin G2 monoclonal antibody that has been developed for the treatment of NMOSD and was approved for first time use in Canada in June of 2020 as monotherapy or as combination therapy in adults and children 12 years or older who are positive for AQP4-IgG autoantibodies (13, 14). Satralizumab was subsequently approved by the US FDA for the same indication on 17 August 2020 (15).

The medication is given subcutaneously and comes in a 120 mg/ml prefilled syringe designed for single use. The currently used dose includes the initial injection of 120 mg, followed by another injection at week 2 and week 4 as loading doses, followed by a maintenance dose of 120 mg every 4 weeks (14). Following the administration of the medication at the recommended dose, the half-life is close to three days and the bioavailability is 78.5% (15).

Although the mechanism of action of Satralizumab is not well understood, it is thought to be mediated through blocking of Interlukin-6 (IL-6) signaling pathways which results in reduction of inflammation and IL-6 mediated autoimmune T- and B-cell activation (16). Satralizumab binds to membrane-bound and soluble IL-6 receptors. This binding results in preventing IL-6 from binding to its substrate and subsequently inhibiting the IL-6 signaling pathways involved in inflammation. The inhibition of T and B cells eventually prevents the differentiation of B cells into AQP4-IgG-secreting plasma cells (16, 17). Due to a unique recycling technology, Satralizumab detaches from IL-6 receptor in a pH-dependent medium which in turn prolongs the duration of circulation in the body (16, 17).

SAkuraStar and SAkuraSky are 2 multinationals, randomized, double-blind, placebo-controlled phase III trials that evaluated the safety and efficacy of Satralizumab in patients with NMOSD (both AQP4-IgG seropositive and seronegative). Briefly, in the SAkuraStar trial patients were randomized 2:1 to receive Satralizumab monotherapy or placebo, while in the SAkuraSky trial, patients were randomized 1:1 to receive Satralizumab add on (in combination with their baseline immunotherapy) or placebo (16). Baseline immunotherapy included azathioprine, mycophenolate mofetil, or oral corticosteroids. For children aged 12 to 17 years at time of enrollment, baseline treatment with azathioprine or mycophenolate mofetil in combination with oral corticosteroid was also permitted (14, 15). Alone or in combination with baseline immunotherapy, Satralizumab was effective in reducing the risk of a judicially considered NMOSD attack (16).

In the SAkuraStar trial, the number of patients who experienced an attack was significantly lower (p = 0.018) in the Satralizumab monotherapy group compared to the placebo group (30% vs 50%). The trial found an attack reduction risk of 55% (16). In the SAkuraSky, a significantly lower number of patients in the add-on Satralizumab group experienced an attack compared with the placebo group (20% vs 43%), with Satralizumab reducing the risk of relapse by 62% (16). While attack rate reduction was shown to be significant in patients who are AQP4-IgG positive, there was insufficient evidence that Satralizumab lowered the risk of NMOSD relapse in patients who were seronegative for AQP4-IgG autoantibodies (17).

Satralizumab was shown to be well tolerated in patients with NMOSD. The trials reported the following adverse effects; headache, arthralgia and injection site reactions (17). In about 2.9% of patients who received the medication, serious adverse events including infection were reported. No anaphylactic reactions or deaths were reported in either of the clinical trials (17).

It is worth mentioning that Tocilizumab which has an off label use might be a safe and effective medication to prevent attacks in patients with NMOSD. In the TANGO trial, Tocilizumab was found to significantly reduce the risk of NMOSD attacks when compared with azathioprine (18) but has a lower level of evidence since the trial was only an open label, multicentre, randomized phase 2 trial.

### Inebilizumab

Inebilizumab is a humanized afucosylated monoclonal antibody that targets CD-19 expressing B cells (19–21). It has been approved by the US FDA in 2020 for the treatment of anti-AQP4 antibody positive NMOSD. It has also been used in several other countries (19–21). It is given as intravenous infusion with 2 initial doses of 300mg separated by 2 weeks and followed by 300 mg every 6 months (21).

B cells have a major role in the pathogenesis of NMOSD. Inebilizumab is a B cell depleting drug that has high affinity to CD-19 expressing B cells and initiates antibody mediated cellular cytotoxicity and cellular phagocytosis. FcRIIIA is a receptor that mediates antibody-dependent cytotoxicity and afucosylation of Inebilizumab increases the affinity by nine-fold to the binding site (19, 21) B cell depleting agents have been studied in the past for management of NMOSD relapses (19–21). Rituximab is one of the medications that has been used off label for treatment of NMOSD. It acts by depleting the CD 20 expressing B cells whereas Inebilizumab acts on CD-19 expressing B cells. CD-19 is also expressed on the pro-B stage of B cells and widely expressed than CD-20. AQP4-IgG antibodies have been produced at various stages of the B cell life cycle and many research studies have focused on CD-19 positive B cells in the management of antibody positive NMOSD (19, 21).

The safety and efficacy of Inebilizumab were evaluated by an international, multicenter, randomized, double-blind, placebo-controlled Phase II/III trial called N-MOmentum study with open-label extension period (19–21). The study included both AQP4-IgG seropositive and seronegative patients. The percentage of seronegative patients was too low for meaningful subgroup analysis. The duration of the randomized control time was about 28 weeks. It was found that at 28 weeks, this medication was effective in reducing the attack rates of NMOSD as monotherapy. During the 28 weeks of treatment, patients treated with Inebilizumab had reduced worsening of the disability with associated fewer lesions on MRI and reduced hospital stay (19–21). Studies have shown that there was a significant reduction of B cells during the treatment. The long-term safety was assessed by the open-label extension period which showed that the effect lasted for more than 4 years (20, 21). Complications like infusion related reactions, arthralgias and infection were similar between the placebo group and Inebilizumab group (19, 20, 22).. Reduced efficacy to vaccination was noted as a consequence of b cell depletion. Inebilizumab also led to a decrease of serum GFAP, as a disease activity biomarker. According to N-MOmentum study, GFAP levels decreased by 12.9% in participants treated with Inebilizumab (21, 23).

## Ongoing clinical trials

Many clinical trials are currently investigating other medications for the efficacy and safety in NMOSD. Some of these medications include the following;

### Belimumab

Belimumab is a monoclonal antibody that has been previously approved for the treatment for use in Systemic Lupus Erythematosus (SLE). The antibody works *via* neutralizing B cell stimulator. A phase II clinical trial is looking at time to relapse in patients with NMOSD while being on monthly Belimumab injections after initial loading. The results are expected in January 2023 (24).

### Bevacizumab

Bevacizumab is a humanized monoclonal antibody that targets vascular endothelial growth factor A. It is currently being evaluated in a drug trial for the treatment in NMOSD. In this trial, a single infusion of bevacizumab is administered in addition to high dose corticosteroids and an additional dose of Bevacizumab is added to PLEX if indicated. The preliminary results have showed promising results that need to be further investigated (25).

### HBM9161(HL161BKN)

HBM9161(HL161BKN) is a human monoclonal antibody which is currently under an open label dose exploration phase 1 trial. HBM9161 acts by blocking neonatal Fc receptor (FcRn) which is an IgG-Fc binding site and accelerates the breakage of IgG and since AQP4-IgG associated with NMOSD is a pathological IgG, it is supposed to be beneficial by rapidly reducing AQP4-IgG levels (26). Of note, FcRn blockade has been proven to be effective in myasthenia gravis (27).

## Conclusion

The recent advancement in our understanding of the pathophysiology of NMOSD has revolutionized the management options and resulted in the introduction of new and safe medications. The newer medications have the ability to target specific elements of the autoimmune cascade in patients with NMOSD. Head-to-head comparisons of (on- and off-label) biological agents for treatment of NMOSD are not available and the differential indication is a matter of ongoing debate. Despite the great success in seropositive patients, the insufficient response in seronegative patients calls for further investigation and more clinical trials.

## Author contributions

All authors listed have made a substantial, direct, and intellectual contribution to the work, and approved it for publication.
